# A simple protocol to produce mature human-induced pluripotent stem cell-derived cardiomyocytes

**DOI:** 10.1016/j.xpro.2021.100912

**Published:** 2021-10-25

**Authors:** Walter E. Knight, Yingqiong Cao, Phoebe Dillon, Kunhua Song

**Affiliations:** 1 Division of Cardiology, University of Colorado Anschutz Medical Campus, Aurora, CO 80045, USA; 2 Gates Center for Regenerative Medicine and Stem Cell Biology, University of Colorado Anschutz Medical Campus, Aurora, CO 80045, USA; 3 Consortium for Fibrosis Research and Translation, University of Colorado Anschutz Medical Campus, Aurora, CO 80045, USA; 4 The University of Denver, Denver, CO 80210, USA

**Keywords:** Cell Biology, Cell culture, Stem Cells, Cell Differentiation

## Abstract

When cultured under typical conditions, human-induced pluripotent stem cell-derived cardiomyocytes (hiPSC-CMs) are structurally and functionally immature. We have previously demonstrated that culture of hiPSC-CMs in maturation medium containing fatty acids, in combination with culture on micropatterned surfaces, produces cells that demonstrate a more mature phenotype compared to standard approaches. Here, we show in detail the steps needed to produce mature hiPSC-CMs. Compared with many approaches, our protocol is relatively simple and can be easily adapted to new laboratories.

For complete details on the use and execution of this protocol, please refer to Knight et al. (2021).

## Highlights

• Two-step protocol to increase maturity of stem cell-derived cardiomyocytes
• Improves sarcomeric organization, contractile function, and metabolic behavior
• Improved cardiomyocyte maturation suppresses baseline hypertrophy
• Simple protocol which most labs should be able to implement

## Graphical abstract

**Figure:**
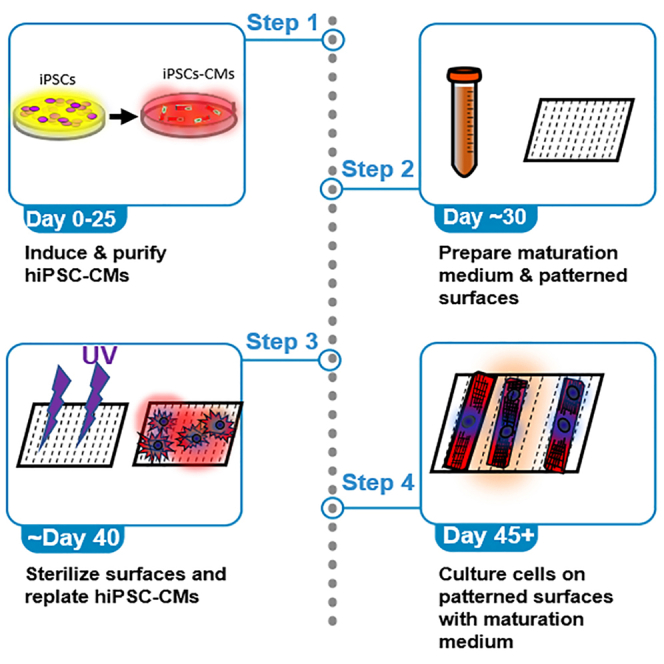

## Teaser

When cultured under typical conditions, human-induced pluripotent stem cell-derived cardiomyocytes (hiPSC-CMs) are structurally and functionally immature. We have previously demonstrated that culture of hiPSC-CMs in maturation medium containing fatty acids, in combination with culture on micropatterned surfaces, produces cells that demonstrate a more mature phenotype compared with standard approaches. Here, we show in detail the steps needed to produce mature hiPSC-CMs. Compared with many approaches, our protocol is relatively simple and can be easily adapted to new laboratories.

## Before you begin

The protocol below describes a method for maturing cardiomyocytes derived from human induced pluripotent stem cells (hiPSCs). While hiPSC-derived cardiomyocytes (hiPSC-CMs) represent a powerful tool for characterizing cardiovascular disease *in vitro*, when cultured under standard conditions, hiPSC-CMs are structurally and functionally immature; in other words, they resemble fetal, rather than adult cardiomyocytes. This fetal phenotype is associated with primarily glycolytic, rather than fatty acid-based, metabolic activity, weak cell and myofibril contractile force, poorly organized sarcomeres, and relatively high cell cycle activity (Birket et al., 2015; Correia et al., 2017; Mills et al., 2017; Pioner et al., 2016; Yang et al., 2014). Additionally, prolonged glucose-based culture induces significant hypertrophy and B-type natriuretic peptide (BNP) expression (Knight et al., 2021). Therefore, using maturation-inducing methods for hiPSC-CM based disease models is crucial. Here, we present a relatively simple method to produce mature hiPSC-CMs, which structurally and functionally recapitulate phenotypes of adult cardiomyocytes to some degree. These methods are relatively simple and require only commercially available reagents and materials. While we briefly describe a protocol to differentiate and select for hiPSC-CMs below, different differentiation protocols may be optimal for different hiPSC lines. We have successfully used this protocol for multiple hiPSC lines; however it should presumably be applicable to any hiPSC line, commercially available hiPSC-CMs, embryonic stem cell line, and possibly for cardiomyocytes derived from non-human iPSC lines as well. However, we will also briefly summarize our hiPSC-CM differentiation and selection protocol below. We prepare hiPSC-CMs via modulation of Wnt signaling, using the method described by Lian et al. (Lian et al., 2012, 2013) and select for cardiomyocytes using the lactate method, as described previously (Tohyama et al., 2013). Recipes for all types of cell media used in this study are also listed in Table 1.

**Table 1:** Reference of constituents of various types of cell media used for this study

Medium type	Basal medium type	Additive 1 /Dilution or concentration	Additive 2 /Dilution or concentration	Notes
RPMI-20 (for replating hiPSC-CMs)	RPMI-1640 with glucose	Fetal bovine serum – 20% (V:V)	Y-27632 (5 μM) – add immediately before use	Store at 4°C for up to 1 month. Y-2 is only necessary when plating cells (not for halting digestion)
RPMI-1640 (for hiPSC-CM culture)	RPMI-1640 with glucose	B-27™ supplement (with or without insulin as specified in the protocol) 1:50 dilution (V:V)		Store at 4°C for up to 1 month.
DMEM with lactate (for hiPSC-CM selection)	DMEM, glucose-free	1M (L+)-lactic acid solution - 1:250 dilution (V:V) – final [] = 4mM		Store at 4°C for up to 1 month.
50**×** (500 mM) galactose solution	RPMI-1640 **without** glucose	Galactose = to final concentration of 500 mM: 45.1 g/500mL		See “step-by-step method details” for solution preparation. Store at −20°C for up to 3 months
Na_2_ EGTA (10mL) - used for myofibril lysis buffer	MQ H_2_O	0.380 g EGTA powder	0.08 g NaOH powder	pH to 7 with KOH. filter with 0.4 μm filter. Store at −20°C for up to 1 year.
1 M K-prop solution(10mL) - used for myofibril lysis buffer	10 mL 1M propionic acid solution	0.561 KOH powder		Mix KOH into propionic acid and filter with 0.4 μm filter. Store at −20°C for up to 1 year.

**Table 2:** 50× fatty acid solution

Reagent	Final concentration	Amount (for 10 mL)
RPMI-1640 **without** glucose	N/A	10 mL
BSA Fraction V (Fatty acid free)	10% (W:V)	1 g
250 mM palmitic acid	2.5 mM (1:100 dilution)	100 μL
500 mM oleic acid	5 mM (1:100 dilution)	100 μL

See “step-by-step method details” for fatty acid conjugation. Store at −20°C for up to 3 months.

**Table 3:** Maturation medium (for hiPSC-CM maturation)

Reagent	Final concentration	Amount (for 500 mL)
RPMI-1640 **without** glucose	N/A	470 mL
50**×** fatty acid solution	1**×** (100 μM oleic acid, 50 μM palmitic acid)	10 mL
50**×** galactose solution	1**×** (10 mM galactose)	10 mL
50**×** B-27™ supplement **with** insulin	1**×**	10 mL

See “step-by-step method details” for solution preparation. Filter w/ 0.22 μm filter before use. Good for 2–3 weeks if stored at 4°C

### Stem cell derived cardiomyocyte differentiation

**Timing: 4–5 weeks before cells will be sufficiently differentiated for maturation methods.**

***Note:*** We prepare hiPSC-CMs via modulation of Wnt signaling, using the method described by Lian et al. (Lian et al., 2012, 2013).

1. Prepare Matrigel-coated plates:
  a. When Matrigel is received, aliquot it such that each individual aliquot is sufficient to produce 6.25 mL of 1**×** Matrigel solution (the exact volume will depend on the lot of Matrigel, and a dilution factor will be provided by the manufacturer). Store Matrigel aliquots at −80°C.
  b. At least two days before plating hiPSCs, thaw out an aliquot of Matrigel overnight (12–18 h) at 4°C.
  c. Working in the sterile hood and keeping Matrigel on ice, dilute it into 6.25 mL cold DMEM/F12 medium. Take care to keep everything cold to prevent Matrigel solution from solidifying.
  d. Add 0.3 mL Matrigel solution per 24 well plate well to be used for induction. Add 6.25 mL solution per 10 cm dish used to grow up hiPSCs.
  e. Wrap plate with paraffin, cover in foil, and store overnight (12–18 h) at 4°C. Plates may be stored in the refrigerator for up to 10 days before use.

2. Plate hiPSCs for induction
  ***Note:*** hiPSC-CM induction, all steps are conducted with 1mL of cell medium per 24 well plate well unless otherwise specified.
  a. Culture hiPSCs of passage 10–30 to 80%–90% confluence on 10 cm Matrigel-coated plates, in mTeSR1™ medium. One confluent 10 cm dish will produce approximately 10 million hiPSCs.
  b. At least one hour before replating cells, remove Matrigel coated 24 well plates from the refrigerator and warm up to room temperature (∼22°C).
  c. Dissociate hiPSCs using 2.5 mL Accumax™ dissociation medium. 3–5 min incubation at 37°C is typically sufficient.
  d. Count cells using hematocytometer and calculate cell yield and density.
  e. Centrifuge cells at 228 *g* and resuspend to a density of 1 million cells per mL in mTeSR1™ supplemented with 5 μM Y-27632.
  f. Plate 375,000 hiPSCs (375 μL) per well onto Matrigel-coated 24 well plate wells (24WPW) and add an additional 0.5 mL medium to each well.
    ***Note:*** This cell number has been found by our lab to be optimal for induction efficiency. Using different hiPSC lines, it may need to be altered to some degree. For the purposes of the induction timeline, this is considered Day -4 (4 days prior to initiating induction).
  g. Culture hiPSCs for 4 days with daily media changes (1 mL mTeSR1™ per well).
    **CRITICAL:** When removing old medium by suction, take care not to scratch off cells from the culture dish.

3. Day 0 (D0): Initiate induction:
  a. Initiate cardiomyocyte induction by changing medium to RPMI-1640 with B-27™ supplement without insulin, supplemented with 6–14 μM CHIR99021 (GSK3β inhibitor).
    **CRITICAL:** For most hiPSC lines in use in our lab, we have identified 8 μM CHIR99021 as optimal for inducing cardiomyocyte induction. However, the optimal concentration will vary by cell line (Lian et al., 2013). We highly recommend optimizing CHIR99021 concentration when inducing cardiomyocytes from a new cell line. Additionally, record the time at which this step was performed, as the next step should be performed exactly 24 hours later.
  b. Exactly 24 h later (D1), change medium to RPMI-1640 with glucose, with B-27™ supplement without insulin.

4. D3: Add combined medium:
  a. Prepare combined medium: from each well of cells, carefully decant 0.5 mL of old medium into 50 mL conical tube. Combine this with an equal volume of fresh RPMI-1640 with glucose, with B-27™ supplement without insulin, and add 5 μM IWP2 (Wnt inhibitor).
  b. Remove the remaining medium from each well, and replace with combined medium.
  c. Repeat this process for each hiPSC line being induced (do not add medium harvested from one cell genotype to cells from another genotype).
5. Continue cell culture:
  a. On D5, change medium to RPMI-1640 with glucose, with B-27™ supplement without insulin.
  b. On D7, and every 3 days thereafter, RPMI-1640 with glucose, with B-27™ supplement with insulin.
6. D15-18: Replate cells: induced hiPSCs typically begin forming beating clusters on D12. Once robust beating is established (usually Day 15–18, large clusters of cardiomyocytes should be visible beating using a low magnification microscope) cells are replated from 24 well plates onto 6 well plates or 10 cm dishes:
  a. The night before replating, coat new cell culture dishes with sterile 0.1% gelatin dissolved in water, overnight (12–18 h) in 37°C cell culture incubator.
  b. Wash cells once with Dulbecco’s phosphate buffered saline (DPBS), and incubate them with 0.25% Trypsin-EDTA for 7 min at 37°C (0.5 mL per well).
  c. Halt digestion via addition of RPMI-1640 medium with glucose, supplemented with 20% fetal bovine serum (FBS) (RPMI-20, 1 mL per well). Pipette cells robustly (approximately 10 times) to detach them, and pipette into a secondary container.
  d. Pellet cells: centrifuge at 228 *g* for 3 min, and resuspend pellets in RPMI-20 supplemented with 5 μM Y-27632.
  e. Plate cells onto gelatin-coated plates (remove gelatin solution first by suction). We typically plate 1∗24WPW onto 1∗6WPW or 4∗24WPW onto 1∗10 cm dish. Use standard media volumes for subsequent culture steps.

***Note:*** If culturing cells under serum-free conditions, RPMI-20 may be replaced with STEMdiff™ Cardiomyocyte Support Medium from Stemcell Technologies. As an alternative to gelatin, induced cardiomyocytes may be plated on fibronectin-coated surfaces (see step 20 for information on coating cell culture dishes with fibronectin) although this will make further replating more difficult, as we have found it is harder to detach cells from fibronectin-coated than from gelatin-coated surfaces. Potentially, other surface coating materials, such as Matrigel® or laminin, may be used, although we have not tried this ourselves.

7. ∼D18-20: Purify cardiomyocytes: 2 days after replating cells. When inducing stem cells into cardiomyocytes, not all cells will assume cardiomyocyte identity. Therefore, it is beneficial to purify cardiomyocytes. This can drastically increase the percentage of cells in culture demonstrating cardiomyocyte identity, producing cultures with greater than 95% Troponin-T positive cells as assessed by flow cytometry (Chi et al., 2018).
  a. Before manipulating cells, prepare 1M lactate solution by diluting 10M L(+)-Lactic acid 1:10 (V:V) in sterile double distilled water.
  b. Prepare lactate medium by adding 1M lactate solution to glucose-free DMEM to final concentration of 4 mM (1:250 dilution by volume)
  c. Filter medium using 0.22 μm filter. Warm medium to 37°C before using.
  d. Wash cells 1**×** with DPBS.
  e. Remove PBS and add lactate medium to cells.
  f. Culture cells in lactate-supplemented DMEM for 5 days, with 1 medium change 2 or 3 days after initiating selection.
  g. After 5 days of lactate selection, replace medium with RPMI-1640 supplemented with B-27™ supplement with insulin. Culture cells in this medium for at least 48 h.
8. ∼D25-27: Continue to culture cells prior to use of maturation methods. At this point, cells may continue to be cultured in RPMI-1640 supplemented with B-27™ supplement with insulin with medium changes twice weekly, or changed to maturation medium, and/or replated onto patterned surfaces. We recommend replating and switching to maturation medium within 40 days of inducing cells to avoid prolonged culture in glucose medium.

## Key resources table

**Table:** 

REAGENT or RESOURCE	SOURCE	IDENTIFIER
**Antibodies**		

Anti-NT-proBNP antibody [15F11] (Use at 1:200 dilution)	Abcam	Ab13115
Anti-sarcomeric alpha actinin (Use at 1:200 dilution)	Millipore Sigma	A7811
Alexafluor 488 goat anti-mouse (Use at 1:400 dilution)	Thermo Fisher Scientific	A11034
Alexafluor 555 goat anti-mouse (Use at 1:400 dilution)	Thermo Fisher Scientific	A21422

**Chemicals, peptides, and recombinant proteins**		

Hoescht 33342 (Use at 1:5000 dilution)	Life Technologies	H1399
Y-27632 dihydrochloride (Rock Inhibitor)	ApexBio	Cat # A3008
CHIR99021 (GSK3 inhibitor)	Cayman Chemical	Cat # 13122
IWP2 (Wnt inhibitor)	Tocris	Cat # 3533
L(+)-Lactic Acid, 90% solution in water (10M)	Acros Organics	Cat # AC 189872500
B-27™ supplement (50**×**) with insulin	Gibco	Cat # 17–504-044
B-27™ supplement (50**×**) without insulin	Gibco	Cat # A18956-01
BSA Fraction V	Goldbio	Cat # A-421-50, Cas 9048-46-8, https://www.goldbio.com/product/6281/bovine-serum-albumin-bsa-fraction-v-fatty-acid-free-for-tissue-culture
D-Galactose	Sigma	Cat # G0750, Cas 59-23-4, https://www.sigmaaldrich.com/US/en/product/sial/g0750?gclid=Cj0KCQjw0K-HBhDDARIsAFJ6UGiwW31tjdcebXhqXgtKOuPUpVlMPXRB8qh1QMPQdRtMfzL9_nuWK7oaAvnKEALw_wcB
Oleic Acid	MP Biomedicals	Cat # 151781, Cas 112-80-1, https://www.mpbio.com/us/oleic-acid-99
D-Galactose	Sigma	Cat # G0750, Cas 59-23-4, https://www.sigmaaldrich.com/US/en/product/sial/g0750?gclid=Cj0KCQjw0K-HBhDDARIsAFJ6UGiwW31tjdcebXhqXgtKOuPUpVlMPXRB8qh1QMPQdRtMfzL9_nuWK7oaAvnKEALw_wcB
Palmitic Acid	Sigma	Cat # P0500, Cas 57-10-3, https://www.sigmaaldrich.com/US/en/product/sigma/p0500
Cellmask™ Orange	Thermo Fisher Scientific	Cat # H32713
Fibronectin	Corning	Cat # 356008
Dry Milk Powder	Research Products International	Cat # M17200
Dulbecco’s Phosphate Buffered Saline (DPBS)	Corning	Cat # 21-031-CV
Triton™-X 100	Sigma	Cat # X100
Fetal Bovine Serum (FBS)	Gibco	Cat # 10437-028
Glucose free DMEM	Gibco	Cat # 11966-025
DMEM/F12	Gibco	Cat # 10565-018
RPMI 1640 media glucose free (for maturation medium)	Gibco	Cat # 11879-020
RPMI 1640 media with glucose	Gibco	Cat # 11875-093
STEMdiff™ Cardiomyocyte Support Medium (for replating cells)	STEMCELL Technologies	Cat # 05027
Accumax™ Cell Detachment Solution	STEMCELL Technologies	Cat # 07921
mTESR1™ Medium	STEMCELL Technologies	Cat # 85850
Matrigel	Corning	Cat # 354277
Gelatin	Sigma	Cat # G2500
32% paraformaldehyde	Electron Microscopy Services	Cat # 15714S
EGTA	Millipore Sigma	Cat # E4378
KOH	Millipore Sigma	Cat # P5958
Propionic acid	Thermo Fisher Scientific	Cat # A258-500
Na_2_SO_4_	Thermo Fisher Scientific	Cat # S379
MOPS	Millipore Sigma	Cat # M1254
MgCl_2_·6H_2_O	Millipore Sigma	Cat # 1374248
ATP	Millipore Sigma	Cat # 3377
Creatine phosphate	ACROS Organics	Cat # AC226790250
Potassium hydroxide (KOH)	Thermo Fisher Scientific	Cat # P250
Sucrose	Millipore Sigma	Cat # S0389
Leupeptin	Tocris	Cat # 1167
Pepistatin A	Tocris	Cat # 1190
Dithiothreitol (DTT)	Millipore Sigma	Cat # 3860-OP
Phenylmethylsulfonyl (PMSF)	Tocris	Cat # 4486
E-64d	Tocris	Cat # 4545
NaN_3_ (sodium azide)	Millipore Sigma	Cat # 247-852-1
Urea	Millipore Sigma	Cat # U5128
Thiourea	Millipore Sigma	Cat # T8656
CHAPS detergent	Millipore Sigma	Cat # CHAPS-RO
EDTA (500 mM solution)	Millipore Sigma	Cat # 324504
Halt™ Phosphatase Inhibitor	Thermo Fisher Scientific	Cat # 78420
Protease Inhibitor Cocktail	Millipore Sigma	Cat # P8340
Tributylphosphine (TBP, 200 mM)	Bio-Rad	Cat # 163–2101

**Experimental models: Cell lines**		

CUSO-1 hiPSC line	Derived by our lab; see Chi et al., (2018)	CUSO-1 hiPSC line. We recommend deriving hiPSC-CMs from passages 10–40.
CUSO-2 hiPSC line	Derived by our lab; see Chi et al., (2018)	CUSO-2 hiPSC line. We recommend deriving hiPSC-CMs from passages 10–40.

**Other**		

Coverslips for patterned surfaces (22 mm squares)	VWR	Cat # 48376-049, https://us.vwr.com/store/product/4646038/vwr-micro-slides-and-coverslips-plastic
Lapping paper for patterning coverslips	Norton Abrasives	Cat # L12F3, 20 micron, https://www.mcmaster.com/4837A111/
Sonicator	Thermo Fisher Scientific	Model FB120 with CL-18 Probe
Bright-Line™ Hematocytometer	Millipore Sigma	Cat # Z359629
6 well plates	Greiner Bio-One	Cat # 657160
24 well plates	Corning	Cat # 353935
10 cm dish	Corning	Cat # 353003
0.2 μm filter	Thermo Fisher Scientific	Cat # FB12566504
0.45 μm filter	Thermo Fisher Scientific	Cat # FB12566503
Tissue-Tearor Homogenizer	Biospec	Model # 985370
Force probes for myofibrils	Self-built; see Woulfe et al. (Woulfe et al., 2019)	N/A

## Step-by-step method details

### Preparation of 50**×** fatty acid solution and 50**×** galactose solution

**Timing: 2 h**

This procedure will describe how to prepare stocks of BSA-conjugated fatty acids and galactose solutions for later preparation of maturation medium. It is essential that fatty acids are conjugated to BSA prior to their use in maturation medium, as otherwise fatty acids will form insoluble droplets or globules which cells will largely be unable to absorb. See Table 1 for the recipe for the recipe for 50X galactose solution, and Table 2 for the recipe for 50X fatty acid solution.

**Figure 1:**
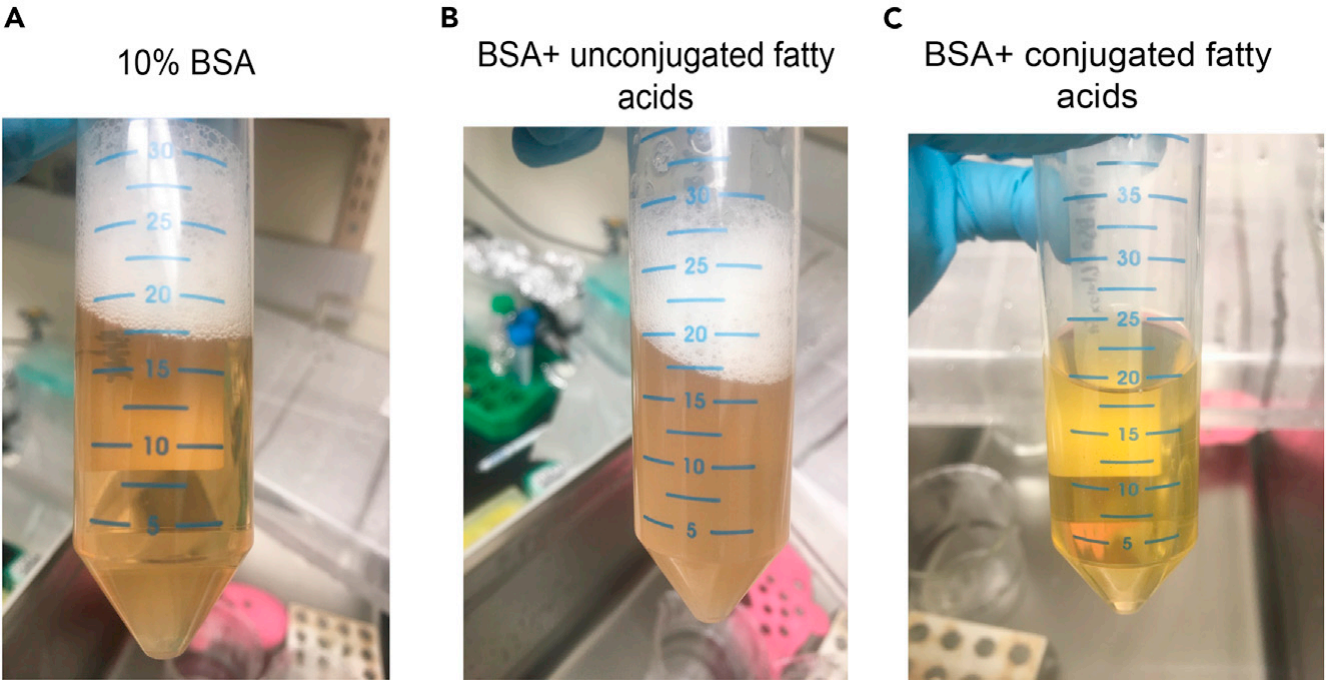
Conjugation of fatty acids with BSA (A) Image of 10% fatty-acid free BSA/glucose-free RPMI solution, with BSA completely dissolved. (B) Image of 10% BSA solution immediately after addition of oleic acid (palmitic acid has already been added and conjugated). Note that the mixture has become cloudy, indicating fatty acid precipitation. (C) Image of the same fatty acid solution after 30 min incubation at 37°C. The solution has cleared, indicating that both palmitic and oleic acid have successfully conjugated to BSA. This solution is ready to be used for preparation of maturation medium (or aliquoted and frozen).

1. Prepare fatty acid stocks:
  a. Prepare a 250 mM palmitic acid solution by dissolving palmitic acid crystals in 100% ethanol, and vortexing until completely dissolved.
  b. Prepare a 500 mM oleic acid solution by diluting oleic acid (oleic acid is liquid at room temperature: (∼22°C) in 100% ethanol.
  c. Fatty acid stocks can be stored, paraffin wrapped, at -20°C for up to three months.

2. Prepare 10% BSA solution:
  a. In a 50 mL conical tube, weigh out 1–4 g of Fatty Acid Free BSA Fraction V (depending on the final volume of 50**×** solution desired).
    ***Note:*** this can be scaled up to produce a larger volume of fatty acid solution, but we do not recommend creating more than 40mL of solution in a single 50 mL conical tube due to BSA frothing that may occur.
  b. In the sterile hood, add glucose-free RPMI to create a 10% BSA solution: for example 10 mL RPMI to 1 g BSA, 20 mL to 2 g, etc.
  c. Vigorously vortex solution until BSA is completely dissolved (Figure 1A). This may require several minutes.
  d. Heat solution to 37°C in water bath for 15 min.

3. Add palmitic acid to BSA:
  a. Add 250 mM palmitic acid solution at a 1:100 dilution, such that the final concentration will be 2.5 mM.
    ***Note:*** Add palmitic acid slowly while agitating solution, as this may reduce aggregation and precipitation of palmitic acid .
  b. Vortex solution vigorously. It is likely that solution will appear somewhat cloudy with fine particles at this point.
  c. Incubate solution for 30 min at 37°C, occasionally vortexing. Solution should shift from cloudy to clear during this process.

4. Add oleic acid solution:
  a. Add 500 mM oleic acid at a 1:100 dilution and vigorously vortex such that the final concentration will be 5 mM. Once again, solution will become somewhat turgid (Figure 1B).
  b. Incubate solution for 30 min at 37°C, occasionally vortexing. Solution should again clear up during this process, as fatty acids conjugate onto BSA (Figure 1C).
5. At this point, the 50**×** fatty acid solution is ready to use.
6. Prepare 50**×** galactose solution:
  a. Weigh out galactose, and resuspend in glucose-free RPMI to a concentration of 500 mM (for example, resuspend 4.51 g galactose in 50 mL RPMI).
  b. Vortex solution vigorously, until galactose is totally dissolved.

**Pause point:** At this point, both 50**×** galactose and 50**×** fatty acid solutions are ready to be used, and may be stored at −20°C for up to three months.

***Alternatives:*** Using Goldbio BSA Fraction V with palmitic and oleic acid, we have always had success conjugating fatty acids to BSA at 37°C. However, if using a different source of BSA, different concentrations or different types of fatty acids, it is possible that a more rigorous conjugation protocol may required. Although this protocol describes specifically conjugating oleic and palamitic acid to BSA for maturation medium, other fatty acids can be used as well – for example, we have also successfully conjugated linoleic acid to BSA. However, depending on the particular fatty acids used, the protocol may need to be modified, in terms of incubation times, temperatures, etc.

### Preparation of maturation medium

**Timing: 30 min**

7. See Table 3 for a complete recipe for maturation medium. Thaw out aliquots of 50**×** galactose solution, 50**×** fatty acid solution, and B-27™ supplement to room temperature (∼22°C).
8. Working in a sterile hood, add galactose solution, fatty acid solution, and B-27™ supplement to glucose-free RPMI medium, each to a 1:50 dilution.
  a. For example, if creating 250 mL maturation medium, add 5 mL 50**×** galactose solution, 5 mL 50**×** fatty acid solution, and 5 mL 50**×** B-27™ supplement each to 235 mL RPMI. This will produce a final concentration of 10 mM galactose, 50 μM palmitic acid, and 100 μM oleic acid.
  b. Mix solution via repeated pipetting or via use of a stir bar until it is completely homogenized.
9. Filter medium using a 0.22 μm filter, into a sterilized/autoclaved container.
  a. Maturation medium preparation is now complete. Medium will be good for 2–3 weeks if stored at 4°C. Medium may also be aliquoted into smaller vessels (under sterile conditions) to avoid repeated warming/chilling cycles.

**CRITICAL:** As fatty acid and galactose solutions are not prepared under fully sterile conditions, it is absolutely essential to filter maturation medium prior to storage or use.

### Prepare patterned coverslips

**Timing: 90 minon day 1; overnight incubation; 2 hon day 2**

This protocol describes the process of preparing and sterilizing micropatterned coverslips for plating of hiPSC-CMs. This entire process is also documented in Methods video S1 of the supplement.

**Figure 2:**
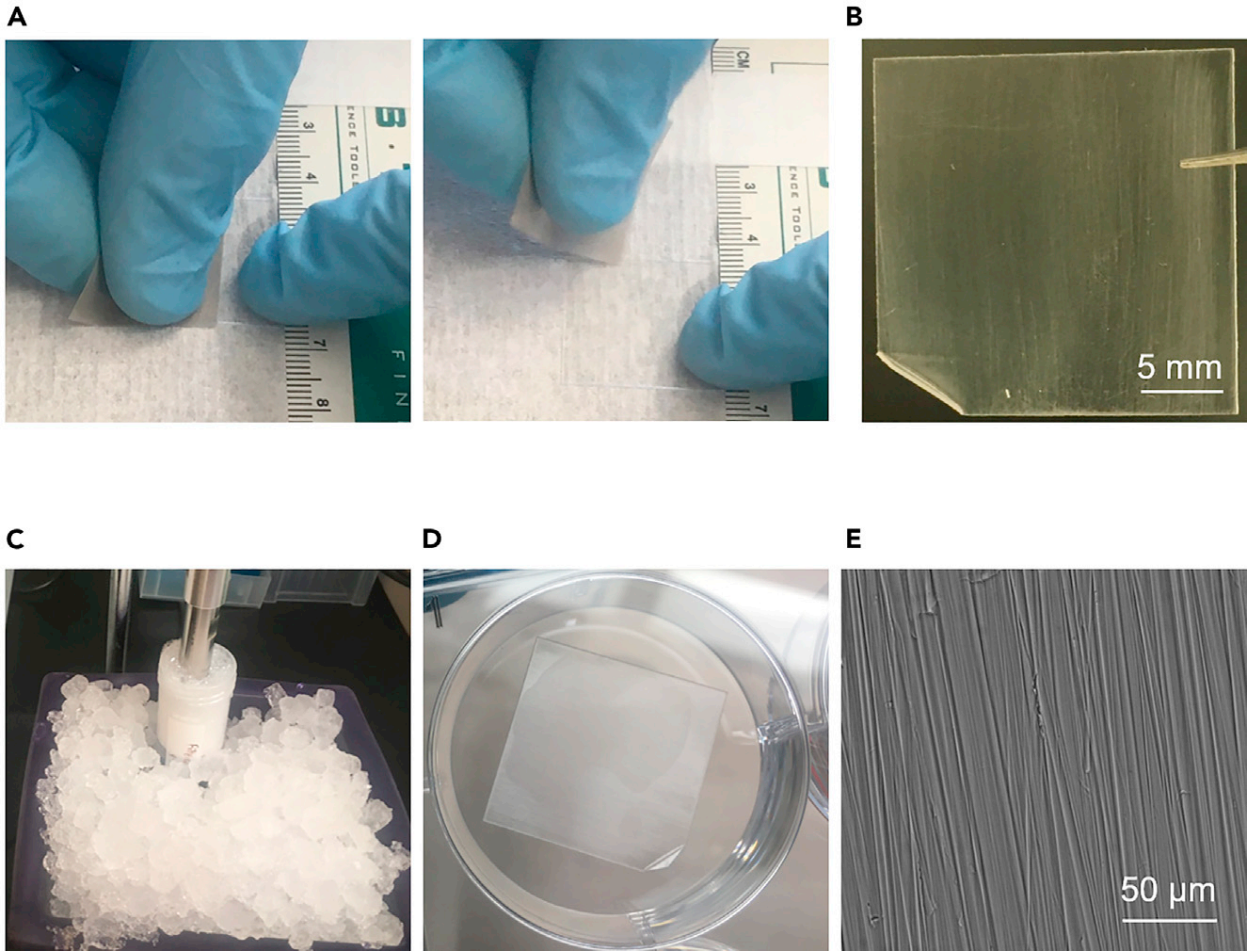
Process of patterning+ sterilizing coverslip (A) Lapping paper being drawn across coverslip to create micropatterns. This is repeated 15–20 times. (B) Closeup image of a patterned coverslip. (C) Photograph of coverslips being sonicated in soapy water to clean. (D) Image of patterned coverslip in a well of a 6 well plate after ethanol evaporation and UV treatment. This coverslip is sterilized and ready for fibronectin coating. (E) Image of patterned coverslip under the microscope.
12. Repeat this process on the other half of the coverslip.
  a. To help ensure linear patterning, it may be useful to use a ruler or straight piece of tape to coordinate coverslip.
  b. After patterning the coverslip, it may also be useful to bend up one corner of coverslip to indicate which side has patterning and allow for later manipulation (Figure 2B). Alternatively, one may consider making a small mark or scratch (such as an X) on the unpatterned side of the coverslip, again to make it easier to identify. This will help prevent mistakenly plating cells on the unpatterned side of the coverslip.
13. Place coverslips in a 50 mL conical tube, and wash twice, with vigorous vortexing, using distilled water. Take care when decanting water between washes to avoid losing coverslips (it may be useful to use mesh or a strainer when decanting water).
14. Incubate coverslips with soapy water. Sonicate them (we use 70% strength, 5 s pulse on, 10 s pulse off, for 3 min total), keeping conical tube on ice to prevent overheating (Figure 2C).
15. Rinse coverslips repeatedly, with vigorous vortexing, with distilled water until soap is completely removed.
16. Incubate coverslips overnight (for 12–18 h) in 100% ethanol on rocker.

***Note:*** The remaining steps are to be conducted in a sterile hood.

**Figure 3:**
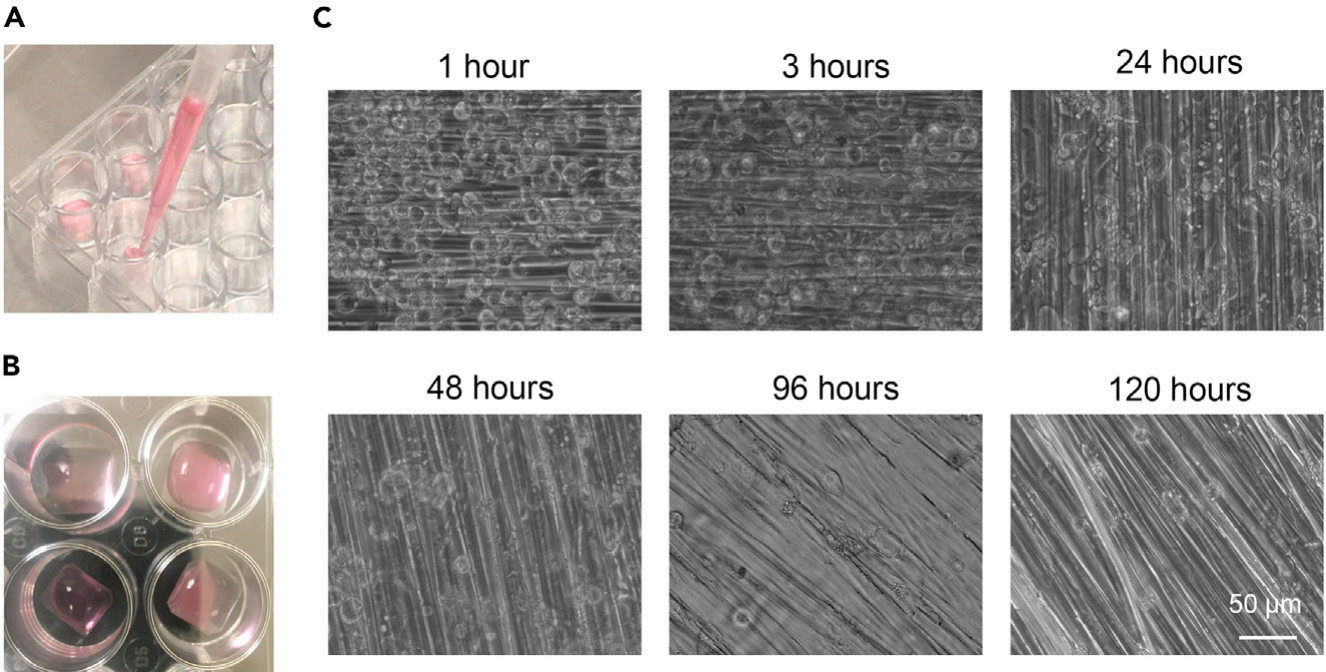
Plating of hiPSC-CMs on patterned surfaces (A) Photograph of hiPSC-CMs being pipetted onto sterilized, fibronectin-coated patterned surfaces. (B) Photograph of patterned surfaces with cell solution for initial cell attachment (prior to filling wells with medium). (C) Microscope images of hiPSC-CMs progressively attaching to surfaces over the first 5 days post plating. In later pictures, cells are difficult to see as they elongate, but can be seen to contract (Methods video S2).
18. Allow ethanol to evaporate from coverslip by leaving them uncovered in sterile hood (this usually takes about 15 min).
19. Incubate coverslips (again, uncovered) under UV light from the sterile hood for 15 min. Coverslips should now be sterile. Make sure that each patterned surface, as well as the remainder of the cell culture containers they are in, are completely dry before proceeding to the next step (Figure 2D).

***Note:*** If one has concerns about whether coverslips have successfully been sterilized, incubate one of them, after sterilization, in antibiotic-free media, without cells, to confirm lack of growth of any microbes.

10. Cut 20 micron lapping paper into ∼2 cm squares.
11. Holding coverslip firmly, draw lapping paper square along half of coverslip 15–20 times. Move lapping paper unidirectionally, in as straight of a line as possible to ensure linear patterning (Figures 2A and 2B).

17. Working in a sterile hood, use forceps to place coverslips in proper size cell culture dishes (we use 6 well plates for full sized coverslips, 24 well plates for quarter sized coverslips) (Figures 2D, 3A, and 3B).

20. Prepare fibronectin solution by diluting fibronectin stock in DPBS, to final concentration of 12 μg/mL. Carefully pipette an equal volume of fibronectin solution onto each patterned surface. The volume chosen should be sufficient to adequately cover the surface, typically about 500 μL per large patterned surface, or 100–125 μL per small patterned surface.
  a. Incubate coverslips with fibronectin solution at room temperature (∼22°C) for at least one hour.
  b. Aspirate all fibronectin solution from coverslips and wash once with DPBS.

**Pause point:** Completely aspirate DPBS. If cell culture plates are wrapped in wrapping film, coverslips may now be stored at 4°C for up to one week. Prior to sterilization and fibronectin coating, patterned coverslips may also be stored for several months at 4°C in ultrapure water.

Methods video S1. A short video demonstrating the construction of patterned surfaces, related to steps 10–12

### Plate cardiomyocytes on patterned surfaces

**Timing: 2 hon day 1; subsequent cell culture can last for weeks to months as desired.**

This protocol describes the process of plating hiPSC-CMs on patterned surfaces and long-term cell culture.

21. Warm up replating and cell dissociation medium to 37°C:
  a. We have used the following medium types for hiPSC-CM replating:
    i. RPMI-20 + Y-2: RPMI medium mixed with 20% (v/v) fetal bovine serum, supplemented with 5 μM Y-27632 (a ROCK inhibitor).
    ii. STEMdiff™ Cardiomyocyte Support Medium (Stem Cell Technologies).
  b. For cell dissociation, we use Accumax™ (Stem Cell Technologies).
  c. Fibronectin-coated patterned surfaces in appropriate cell culture containers should also already be prepared and on hand.
22. We recommend replating hiPSC-CMs between day 35 and 45 post induction. Cells should be plated on sterile 0.1% gelatin-coated dishes and grown to a high degree of confluency prior to replating, as detailed earlier in this protocol.
23. Wash cells once with room temperature (∼22°C) DPBS.
24. Add Accumax™ or other dissociation medium to cells (typically 2.5 mL for a 10 cm dish, 0.5 mL for a 6WPW).
  a. Incubate cells at 37°C for 5–9 min.
  b. Observe cells during dissociation until cells begin to detach. You may agitate cell culture dishes and tap the bottom of the cell culture dish lightly to help promote cell detachment.
25. Once cells have detached, halt dissociation by adding an equal volume of cell replating medium (RPMI-20 + 5 μM Y-27632 or STEMdiff™ Cardiomyocyte Support Medium) to dissociated cell mixture.
  a. Pipette medium up and down several times to help detach cells and create single cell suspension.
  b. Cell detachment should be visually apparent but it may be helpful to confirm by observing cell culture dishes under a microscope.
  c. Pipette cells into a 10 or 50 mL conical tube (depending on volume of cell solution). It may be beneficial to wash plates with additional medium to ensure all cells are transferred.
26. Count cells using hematocytometer, and calculate cell concentration and total yield.
27. Carefully aspirate medium from cell pellet, and resuspend in replating medium, to density of 500,000 to 1,000,000 cells/mL. Gently pipette cells up and down repeatedly to further homogenize cell suspension.
28. Carefully add cell suspension onto patterned surfaces, dropwise. Try to slowly cover the entire surface with cell solution (Figure 3A).
  a. For large surfaces (6WP), add 300k–500 k cells/surface. For small (24WP) surfaces, add 30k–60 k cells per surface.
    i. This plating density will generally yield a confluent hiPSC-CM monolayer. Lower densities may be preferable for microscopy of isolated cells.
  b. If cell suspension added doesn’t adequately cover patterned surface, slowly add additional media across surface (again, dropwise) but do so very slowly to avoid causing cell solution to run off of surface.

**CRITICAL:** Do not add excess media to patterned coverslips, or cells and solution will run off of coverslip into well. Be extremely careful handling cell culture dishes containing coverslips. If this occurs, it may be possible to salvage the well by pipetting up excess solution, then using vacuum suction to ∗thoroughly∗ dry the area around the coverslip, then adding cell solution back onto coverslip. Plating cells at a high density reduces the volume of solution which must be added per coverslip, thus reducing the likelihood of runoff.

29. Allow cell solution to sit on patterned surface for at least 30 min before adding additional medium, to allow hiPSC-CMs to attach to the coverslip (Figure 3B).
  a. After this, bring medium volume up to typical amount using replating medium (aka 2 mL for 6WPW, 0.5 mL for 24WPW).
  b. Over the next several hours, cells should begin to elongate as they spread out along surface (Figure 3C). As cells attach and elongate, they will become more difficult to see.
  c. When plated at a high density, hiPSC-CMs will generally form a monolayer exhibiting synchronous contraction along the direction of patterning (Methods video S2).
30. 48 h after replating cells, switch cells to maturation medium. Cells may be cultured in maturation medium indefinitely depending on desired cell age at time of experimentation.
  a. When changing medium, remove old medium via suction from an area of the cell culture well outside of the patterned surface to avoid scratching off cells. As with any cell culture, work quickly when changing medium to avoid drying out cells.
31. When cells have been cultured for the desired duration, they may be fixed and stained (see below), harvested for RNA or protein studies, or other protocols in a similar fashion as hiPSC-CMs cultured via other approaches.
  a. When lysing cells, it will be necessary to use vigorous scraping to detach cells from surfaces.

***Note:*** We typically culture cells on patterned surfaces until day 55–75 post induction: this duration of culture is typically sufficient to produce elongated cardiomyocytes with relatively organized sarcomeres and low BNP expression. We have cultured cells up to day 120 post induction, but observed relatively few changes in terms of gene expression or myofibril mechanics compared to day 75 (unpublished observations).

Methods video S2. A synchronously contracting hiPSC-CM monolayer, cultured on a patterned surface in maturation medium, related to steps 30 and 31

### Fix and stain hiPSC-CMs plated on patterned surfaces for BNP

**Timing: 2 days: approximately 2 hof work on each day.**

This protocol describes the process of fixing hiPSC-CMs and staining them for the hypertrophic marker BNP. Cells may then be stained for other markers and imaged. The protocol for staining for BNP is immunofluorescence-based and is adapted from a previous protocol (Carlson et al., 2013).

32. Prepare permeabilization buffer: 3% (w/v) milk and 0.1% (v/v) Triton-X 100 in DPBS. Use this solution within 5 days of preparation and store at 4°C. This step can be delayed if storing fixed cells before staining.

33. Fix cells:
  a. Wash cells to be fixed once with DPBS.
  b. Fix cells in 4% paraformaldehyde/PBS for 15 min at room temperature (∼22°C).
    ***Note:*** Although lab-prepared paraformaldehyde solution is acceptable for staining cells, we have had best results when using commercially available, electron microscopy-grade paraformaldehyde (see key resources table). For other stains, other fixatives may also be used, but methanol-acetone may change the color of (bleach) the plastic of the coverslips.
  c. Wash cells once with DPBS.
    **Pause point:** After fixing cells and removing paraformaldehyde solution, fixed cells may be immersed in DPBS and stored in wrapping film sealed containers at 4°C for 1–2 weeks.

34. Permeabilize cells: incubate patterned surfaces (coverslips) with permeabilization buffer at room temperature (∼22°C) for 30 min.
35. Incubate cells with primary antibody: dilute BNP antibody (in our case, Abcam ab13115, 1:200 dilution) in permeabilization buffer, and add over coverslips.
  a. Incubate coverslips with primary antibody solution overnight (12–18 h) at 4°C. We recommend performing this incubation in a wrapping film or Saran wrap-sealed container, to prevent evaporation and minimize volume of antibody solution required.
36. The following day: wash cells;
  a. Wash coverslips 3 times with DPBS supplemented with 0.05% (v/v) Triton-X 100. Incubate cells at 5 min at room temperature (∼22°C).
37. Add secondary antibody solution, diluted in permeabilization buffer.
  a. We typically use Alexa Fluor 488 secondary goat anti-mouse antibody, diluted 1:400, for BNP staining, but other antibody types should work as well.
  b. Incubate coverslips in secondary antibody solution at room temperature (∼22°C) for 1 h.
38. Wash cells:
  a. Wash coverslips 3 times with DPBS supplemented with 0.05% (v/v) Triton-X 100. Incubate cells at 5 min at room temperature (∼22°C).

***Optional:*** Counterstain cells for other markers as desired. While we have found that optimal BNP staining can be achieved using milk-based permeabilization buffer as indicated, most other markers, including sarcomeric proteins, stain well using more typical methods, such as blocking and antibody dilution solutions based on horse or goat serum in DPBS, or using DAKO reagents. These stains can be performed after staining for BNP, without any noticeable loss of BNP signal.

**Figure 4:**
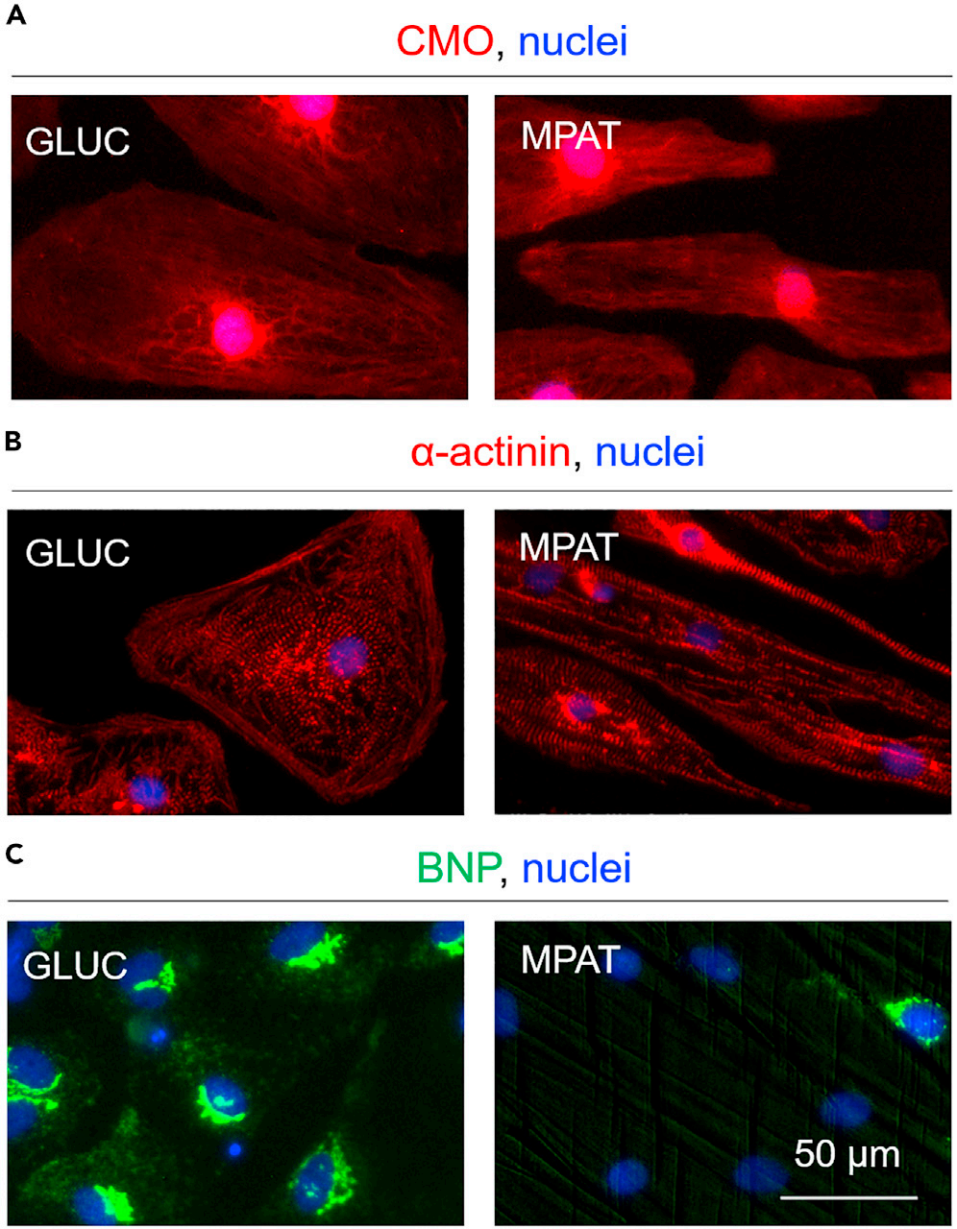
Morphological changes induced in hiPSC-CMs with maturation-inducing culture (A) Cellmask™ Orange-stained (CMO) hiPSC-CMs cultured in standard glucose-based RPMI (GLUC), or in maturation medium on patterned surfaces (MPAT). (B) α-actinin staining in hiPSC-CMs. The patterned hiPSC-CMs demonstrate significant elongation, with a greater proportion of α-actinin localized perpendicular to the long axis of the cells. (C) BNP/pro-NT-BNP staining in hiPSC-CMs. Culture with maturation medium and on patterned surfaces suppresses BNP expression.

39. Stain nuclei and/or mount coverslips:
  ***Note:*** If using mounting medium containing DAPI, it is unnecessary to conduct nuclear staining.
  a. If staining cells directly, dilute a nuclear dye such as DAPI or Hoescht 33342 1:5000 in DPBS, and incubate coverslips with this solution for 5 min at room temperature (∼22°C).
  b. Wash cells twice with DPBS.
  c. Mount coverslips: on a glass slide, place a small drop of mounting medium such as Vectashield® or Prolong™ Gold, and place coverslip with the patterned side facing down into mounting medium.
    ***Note:*** Gently press onto coverslip to remove any bubbles. Remove excess mounting reagent by vacuuming or pipetting.
    **CRITICAL:** Make absolutely sure that the cell covered/patterned side of the surface is in mounting medium here (facing down) as otherwise cells will rapidly dry out and signal will be lost.
  d. Seal coverslip to slide using nail gloss. Cells are now ready to image.
  e. If staining is successful, BNP is primarily observed to be localized proximal to cell nuclei. Please note that maturation medium suppresses BNP expression, so relatively few BNP positive cells will be observed at baseline, in the absence of hypertrophic agonists (Figure 4C). Some degree of autofluorescence will likely be observed on patterned surfaces, especially in the green channel.

### Isolate myofibrils from hiPSC-CMs

**Timing: 1 h to isolate myofibrils, several additional hours if measuring myofibril mechanics. Extensive solution preparation required before beginning.**

**Figure 5:**
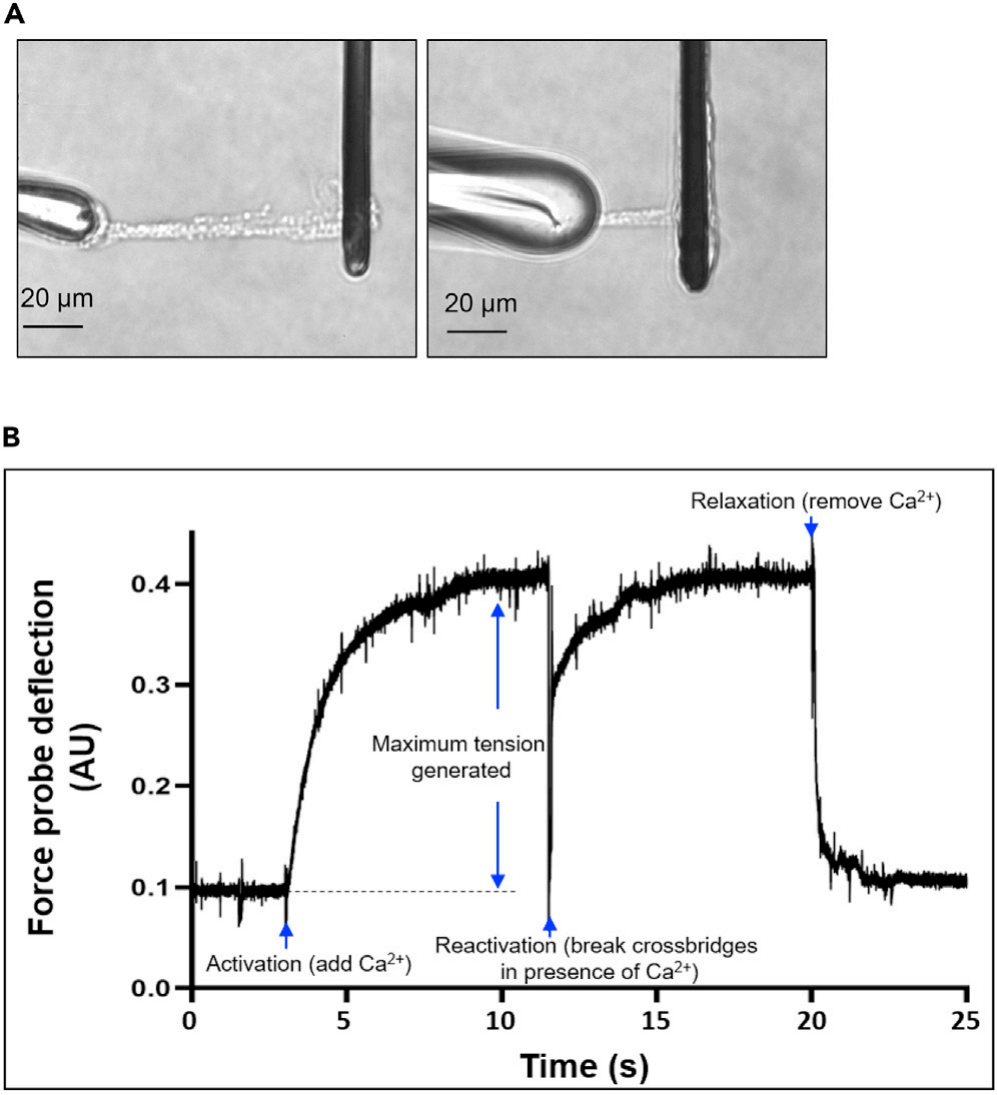
Myofibrils and a representative myofibril activation/relaxation trace (A and B) (A) Myofibrils isolated from hiPSC-CMs cultured under MPAT conditions, and mounted between glass microtools in relaxing solution. Different sizes of stretchers, the microtool on the left, may be used, as seen in (A and B). When Ca^2+^ is added to this solution, the myofibril will contract, pulling the force probe (the dark microtool) to the left. When Ca^2+^ is removed from solution, the myofibril will relax. By tracking the deflection of the force probe, which is displayed on the y-axis, an activation/relaxation trace as show in (B), can be prepared. This trace was also derived from a myofibril from an MPAT hiPSC-CM. From such a trace, speed of activation and relaxation, as well as force generated by the myofibril, can be calculated. Please note that significant equipment and protocols which are not covered by this manuscript will be needed to fully conduct myofibril mechanics experiments.

***Note:*** This protocol describes ONLY how to isolate myofibrils from hiPSC-CMs. These myofibrils can then be used for protein studies, or for measuring myofibril mechanics. The protocols and equipment needed to measure mechanics are quite sophisticated and specialized and therefore will not be described here – for further information on these techniques, please refer to Woulfe et al. (Woulfe et al., 2019). For examples of isolated myofibrils from cells cultured in maturation medium and on patterned surfaces (MPAT) and an activation/relaxation trace from a myofibril, see Figure 5. Preparing bath and myofibril lysis buffer takes some time. Prepare these before beginning this procedure.

40. For characterizing myofibril mechanics, we recommend using 2 full size (22 mm by 22 mm) patterned surfaces with a confluent layer of hiPSC-CMs (typically 350k–500 k cells per patterned surface) per experiment.
41. Prepare myofibril lysis buffer as described in Tables 4 and 5. Cool this buffer on ice.

**Table 4:** Myofibril bath buffer (Stock)

Reagent	Final concentration	Amount (for 100 mL)
Na_2_EGTA (100 mM, pH 7)	10 mM	10 mL
K-prop (1 M)	54 mM	5.4 mL
Na_2_SO_4_ (100 mM)	17.69 mM	17.69 mL
MOPS (1M)	10 mM	1 mL
MgCl_2_ (1M)	6.08 mM	608 μL
ATP∗	6.70 mM	0.3396 g
Creatine phosphate∗	15.5 mM	0.327 g

To prepare, mix the first 5 ingredients, pH solution to 6.8, add final two starred components (ATP and creatine), and pH solution to 7, and filter with 0.45 μm filter. Store at −20°C for up to 1 year.

**Table 5:** Myofibril lysis buffer (Stock)

Reagent	Final concentration	Amount (for 5 mL)
Myofibril bath	N/A	5 mL
Sucrose	0.584 M/ 20 % W:V	1 g
Leupeptin	10 μM	5 μL 10 mM solution
Pepistatin A	5 μM	12.5 μL 2 mM solution
Dithiothreitol (DTT)	1 mM	10 μL 500 mM solution
Phenylmethylsulfonyl (PMSF)	200 μM	10 μL 100 mM solution
E-64	10 μM	5 μL 10 mM solution
NaN_3_ (sodium azide)	500 μM	2.5 μL 1 M solution

To prepare, mix all ingredients immediately before use. Store once ice until used to lyse cells. Do not store long term. In steps 46–47, to wash myofibrils, use myofibril lysis buffer with all components **except** sucrose.
42. Wash hiPSC-CMs once with DPBS. Remove PBS using vacuum.
43. Add chilled myofibril lysis buffer to each patterned surface to be lysed.
  a. Using a plastic scraper, lift cells from patterned surfaces. Vigorously scrape cells, passing scraper over patterned surface 20 or more times. Cells detaching from surface should be visible (lysis buffer will become increasingly cloudly).
  b. Pipette cell lysate into 1.5 mL microfuge tube. Vortex solution vigorously.
44. Incubate lysate on ice for 10 min, periodically vortexing.
45. Centrifuge lysate at 1500 *g* for 5 min, at 4°C.
  a. After centrifugation, a small myofibril-rich pellet should be visible.
46. Remove supernatant, and wash pellet with fresh myofibril bath with protease inhibitors (same as in step 42, but without sucrose; see Table 5).
47. Repeat steps 45 and 46 twice more, to remove any residual sucrose.
48. Homogenize lysate using a brief (∼5 s) pulse of a Tissue Tearor Homogenizer at 30–50% of maximum speed. Avoid introducing bubbles into solution. Under the microscope, elongated myofibrils should now be visible, along with cell debris. The lysate is now ready for use in mechanical studies.
49. If using myofibrils for protein studies, centrifuge solution at 1500 g for 15 min to pellet myofibrils. Remove relaxing solution, and resuspend myofibrils in isoelectric focusing buffer (IEF, see Table 6). Lysate may be frozen at −80°C until use.

**Table 6:** Isoelectric focusing solution (IEF)

Reagent	Final concentration	Amount (for 5 mL)
Urea	8.07 M	2.425 g
Thiourea	2.5 M	0.95 g
CHAPS	4 % (W:V)	200 mg
EDTA	2 mM	20 μL 500 mM solution
Dithiothreitol (DTT)∗	10 mM	100 μL 500 mM solution
Tributylphosphine (TBP)∗	2 mM	50 μL 200 mM solution
Sigma P8340 Protease Inhibitor Cocktail∗	1**×**	50 μL 100 solution
Halt™ Phosphatase inhibitor∗	1**×**	50 μL 100**×** solution

To prepare, mix first four ingredients and filter with 0.45 μm filter. Aliquot and store for up to 1 year at −20°C. Immediately before use, thaw aliquot of solution, vortex to clear out crystals and add final four starred ingredients. Usually only a small volume (1 mL or less) of IEF will be used for a single experiment, so scale down these ingredients accordingly. Keep IEF with inhibitors on ice until use, but do not store long term.

## Expected outcomes

hiPSC-CM culture with patterned surfaces and using maturation medium will produce hiPSC-CMs which morphologically (Figures 4A and 4B) and functionally demonstrate an increased degree of maturity, compared to hiPSC-CMs cultured in standard glucose-based medium. Typically, this involves a greater degree of cell elongation and sarcomere organization (Figures 4A and 4B), reduced expression of the hypertrophic marker B-type natriuretic peptide (BNP, Figure 4C), and increased sarcomere organization, contractile force, and reduced cell area with prolonged culture (Knight et al., 2021), shifting these cells towards a more adult-like phenotype.

## Limitations

Limitations: Compared to standard culture, cell yields from patterned surfaces are relatively low (typically approximately 50% of that of standard culture, on a well per well basis). This may be due to the smaller area of patterned surfaces, cells not attaching to the entire area of surfaces, and that hiPSC-CMs cultured in fatty acid-based medium (such as maturation medium) essentially cease dividing (Mills et al., 2017). Accordingly, we recommend that experiments be planned accordingly, scaling up patterned surface culture as necessary to ensure that sufficient numbers of cells are attained. While the cells produced by this method will be much more mature than hiPSC-CMs derived via standard glucose-based culture, they do not fully resemble or recapitulate the function of adult cardiomyocytes.

## Troubleshooting

### Problem 1

Occasionally, we have noticed that cells attach poorly to patterned surfaces, resulting in low yields (step 28).

### Potential solution

To some degree, this appears to depend on the batch of hiPSC-CMs used: it may be useful to repeat using a different batch of cells. Otherwise, consider increasing the amount of time hiPSC-CMs are incubated on surfaces before adding additional medium (step 30), as this may wash cells off of surfaces. Switching cells to maturation medium too quickly after replating also appears to stress cells and may cause cell detachment or death. In this case, it may be beneficial to culture cells for 1–2 days in RPMI-1640 supplemented with B-27™ (with glucose) prior to switching cells to maturation medium.

### Problem 2

When staining hiPSC-CMs for cardiac markers, many cells stain negative for these markers, or similarly, when culturing cells on patterned surfaces, cells on large areas of the surface do not beat (steps 30 and 31).

### Potential solution

It is likely that insufficiently stringent selection for cardiomyocytes was used. If many nonmyocytes survive selection and are plated on patterned surfaces, they will attach to the surfaces and continue to divide, eventually outcompeting/replacing most cardiomyocytes. This is exacerbated by the tendency of maturation medium to induce cell cycle arrest. If this occurs, it will be necessary to conduct a new hiPSC-CM induction, and it may be beneficial to culture cells for more than 5 days in lactate medium, although this will eventually begin to kill cardiomyocytes as well.

### Problem 3

hiPSC-CMs become contaminated after plating on patterned surfaces (step 28, patterned surface sterilization detailed in step 16–19).

### Potential solution

It is very likely that surfaces are being insufficiently well sterilized. Consider increasing duration of incubation in 100% ethanol, or duration of exposure to UV light. Confirm that ethanol is completely evaporated before UV light sterilization. Consider flipping patterned surfaces over during UV sterilization to sterilize bottom surface of surfaces (although we have never found this to be necessary).

### Problem 4

hiPSC-CMs become contaminated after switching to maturation medium (step 30).

### Potential solution

It is likely that contaminants are present in maturation medium. Filter media through sterile, 0.22 μm filter prior to use of media, and conduct filtration in a sterile hood. Consider double filtering media before use. If nothing else works, one may consider supplementing maturation medium with antibiotics, although this could potentially have deleterious effects on cardiomyocyte function (Belus and White, 2001).

### Problem 5

Fatty acids precipitate out of 50**×** fatty acid solution during preparation (typically the precipitate will take the form of fine white dots or possibly crystals) (steps 3 and 4).

### Potential solution

Fatty acids are being insufficiently well conjugated to BSA. We would recommend initially increasing incubation time at 37°C. If this does not solve the problem, consider increasing the incubation temperature, up to 50°C. It was also be beneficial or necessary to sonicate the BSA/fatty acid mixture.

## Resource availability

### Lead contact

For further information or to request reagents, please direct requests to Dr. Kunhua Song, kunhua.song@cuanschutz.edu.

### Materials availability

The materials used for this study can be created from commercially available materials, as is indicated in Table 1.

## Data Availability

No data sets or code were generated in the process of conducting this study.
